# Gene co-citation networks associated with worker sterility in honey bees

**DOI:** 10.1186/1752-0509-8-38

**Published:** 2014-03-26

**Authors:** Emma Kate Mullen, Mark Daley, Alanna Gabrielle Backx, Graham James Thompson

**Affiliations:** 1 The University of Western Ontario, 1151 Richmond Street North, London, ON N6A 5B7, Canada; 2 Ontario Veterinary College, University of Guelph, 411 Gordon Street, Guelph, Ontario N1G 2W1, Canada

**Keywords:** *Apis mellifera*, Co-citation network, Gene expression, Hub gene, Reproductive altruism, Ovaries, Pheromone

## Abstract

**Background:**

The evolution of reproductive self-sacrifice is well understood from kin theory, yet our understanding of how actual genes influence the expression of reproductive altruism is only beginning to take shape. As a model in the molecular study of social behaviour, the honey bee *Apis mellifera* has yielded hundreds of genes associated in their expression with differences in reproductive status of females, including genes directly associated with sterility, yet there has not been an attempt to link these candidates into functional networks that explain how workers regulate sterility in the presence of queen pheromone. In this study we use available microarray data and a co-citation analysis to describe what gene interactions might regulate a worker’s response to ovary suppressing queen pheromone.

**Results:**

We reconstructed a total of nine gene networks that vary in size and gene composition, but that are significantly enriched for genes of reproductive function. The networks identify, for the first time, which candidate microarray genes are of functional importance, as evidenced by their degree of connectivity to other genes within each of the inferred networks. Our study identifies single genes of interest related to oogenesis, including *eggless*, and further implicates pathways related to insulin, ecdysteroid, and dopamine signaling as potentially important to reproductive decision making in honey bees.

**Conclusions:**

The networks derived here appear to be variable in gene composition, hub gene identity, and the overall interactions they describe. One interpretation is that workers use different networks to control personal reproduction via ovary activation, perhaps as a function of age or environmental circumstance. Alternatively, the multiple networks inferred here may represent segments of the larger, single network that remains unknown in its entirety. The networks generated here are provisional but do offer a new multi-gene framework for understanding how honey bees regulate personal reproduction within their highly social breeding system.

## Background

The well-understood theory of kin selection explains how complex social behaviour can evolve at the gene level [1-3], yet the theory does not predict which genes promote the expression of reproductive altruism. The recent genome sequencing of the honey bee *Apis mellifera*[4] and of other eusocial organisms (e.g. [5,6]), is creating new opportunities to identify genes involved in reproductive regulation and social coordination. For example, *vitellogenin*[7,8], *major royal jelly proteins*[9], insulin signaling genes [10,11], and ecdysteroids [12,13] are among a growing set of genes implicated in reproductive regulation. Despite these advances from microarray and quantitative PCR studies, there has not yet been an attempt to link these genes into functional pathways that explain the phenotypic expression of worker sterility.

Honey bees are a model system for studying the sociogenomic basis of worker reproductive altruism and sterility [4,14,15]. Like other highly social taxa, eusociality in honey bees is characterized by a reproductive division in labour between reproductive and non-reproductive specialists [16]. The queen caste is sexual and highly fecund, with well-developed ovaries that each contain ~150-180 ovarioles. The worker caste, by contrast, is non-sexual and has only rudimentary ovaries with few ovarioles [17]. Workers are effectively sterile in the presence of a functional queen, and though this trait has many physiological components, sterility is most commonly measured as a function of ovary activation [18]. One approach to identifying genes integral to the expression of worker reproductive altruism and sterility is therefore to screen for genes that control ovary activation [9].

For workers, sterility from ovary inactivation is not obligate but rather is conditional on social context. As predicted from kin theory, workers refrain from activating their ovaries to lay eggs when the indirect fitness pay-off surpasses a conditional threshold [19]. For individual workers this threshold is in part dependent on queen fecundity, and is communicated to workers by the queen’s pheromonal signal [20,21]. When a queen is healthy and fecund, her daughter workers will generally refrain from activating their ovaries, but when she is weak or absent, a proportion of workers may activate their normally dormant ovaries to lay unfertilized eggs [22]. Because worker sterility is conditional on the strength of queen signal, we likewise expect genes regulating ovary activation to be conditionally expressed – in particular, in response to queen mandibular pheromone (QMP).

Previous studies have begun to identify genes differentially expressed as a function of pheromone [8,9,11,23-25], but as yet no study has systematically compared these gene lists or compiled them into a network of potentially interacting genes that collectively function to turn worker ovaries *on* and *off*[12,26]. Inferring a gene network for the control of worker ovary activation will help determine how worker sterility is regulated at the molecular level, and will represent our best example yet of how genes interact with each other and with their environment to coordinate one of the best-known forms of reproductive altruism.

Using a network biological approach [27], we first collect studies from the literature that identify genes differentially expressed by workers as a function of queen signal. Second, from comparable studies we infer, for the first time, the functional relationship among candidate genes using co-citation networks. A co-citation network is a graphical representation of how genes interact with each other to functionally affect a phenotype. The graphs infer pairwise interactions between genes if they are mentioned within the same sentence of a written abstract published in PubMed – the co-citation being used to suggest a functional relationship between them [28].

Candidate genes identified from microarray studies alone are typically those with the highest or most consistent expression differences. Network analysis, by contrast, builds upon these gene-list outputs to identify genes of importance via a different criterion – namely, those with the highest connectivity [29]. Identifying well-connected ‘hub’ genes within networks can help pinpoint the crucial junctures that enable network function [30]. Given the flurry of gene expression analyses that proceeded the Honey Bee Genome Sequencing Project e.g. [31-33], there is now worldwide interest in converting the data generated from these analyses into provisional networks that describe how worker sterility is regulated within eusocial bee colonies. Moreover, the as-yet-unknown network is potentially related to the networks that regulate other aspects of honey bee social coordination, such as a tendency to specialize on pollen vs. nectar among foraging workers [34,35], or the tendency for individual workers to specialize on within-colony vs. out-of-colony tasks [26,36].

For honey bees, several studies have suggested a single, conserved pathway that regulates ovaries in response to pheromonal cues [8,9,11,37]. In this study we test this single-pathway hypothesis by generating co-citation networks from genes previously implicated in the regulation of worker ovaries. First, we identify suitable microarray experiments that derive gene sets related to ovary activation. We then use the computer software suite Genomatix Pathway System (Genomatix, Munich) to evaluate whether co-citation networks can adequately explain variation in this trait. From the networks inferred, we test whether worker ovary activation is best explained by a single, conserved pathway that is retrieved by different studies, or whether variation in this trait is better explained by multiple networks that vary with regard to the age, population or pheromone treatment of workers. This latter scenario would suggest that no single pathway explains the conditional expression of worker sterility, and that multiple pathways are utilized by workers under different circumstances, in different populations or at different phases in a worker’s life. Finally, our analysis will allow us to test the extent to which any inferred networks show homology to those known from *Drosophila* or other insects, as predicted from recent sociogenomic theories [35,38].

## Methods

### Meta-analysis and network construction

In October 2012, we compiled microarray data from the literature by searching the Web of Science using the following search criteria: [TOPIC = honey bee OR honeybee OR *Apis mellifera*] coupled with [TOPIC = gene expression OR microarray] and [TOPIC = steril* OR ovar*], whereby the latter terms capture topics such as sterile, sterility, ovary, ovarian, etc. We also searched for analyzed microarray data directly using the search function of ArrayExpress online databases (http://www.ebi.ac.uk/arrayexpress) with the filter [Species = *Apis mellifera*].

To circumscribe studies that most closely identify genes that regulate ovary activation, we included data sets from studies that met the following criteria. Studies must have i) reported normalized gene-expression differences between ovary-active and ovary-inactive adult workers, ii) controlled for genetic and environmental background through standardized rearing conditions, iii) used queen mandibular pheromone [39] as the principle cue for manipulating ovaries, and iv) quantified the level of ovary activation via an explicit scoring scheme. Studies that were generally on-topic but that did not have a pheromone-untreated control group [24], did not use queen mandibular pheromone [12,25] or did not explicitly score ovaries [25] are valuable in their own right but were excluded from our meta-analysis.

Prior to up-loading acquired microarray data into Genomatix Pathway System, we first generated standardized gene lists. This pre-processing step enabled comparison between studies that used different sets of microarray probes. For studies using the complimentary DNA (cDNA) platform described in Whitfield et al. [40], we converted expressed sequence tag (EST) accessions to the corresponding gene accession from Version 2.0 of the Official Gene Set [41]. We then manually curated and identified the single best significant (*E*-value < 10^-5^) BLASTp match in *Drosophila melanogaster* [Version 5.10; [42]]. Bee ESTs that did not correspond to a coding sequence in the fly were, out of necessity, excluded from downstream analysis (a minority of genes, see Results). For studies using the honey bee oligonucleotide microarray, in which probes are already linked to the Official Gene Set (array described at ArrayExpress under accession A-MEXP-755), we simply used BLASTp to directly assign the most likely *D. melanogaster* homologue. The meta-data matrices that we used as input for pathway analysis therefore consisted of fly homologues that correspond to the differentially expressed bee genes. The database and pathway analysis algorithms of Pathway System software are optimized for the fly and we simply transferred the direction and magnitude of bee gene expression changes to the fly homologues. We uploaded gene lists and expression profiles to Genomatix Pathway System. The algorithm uses a gene recognition strategy described by Frisch et al. [43] to scan the PubMed database for genes mentioned together and it subsequently builds the network by adding interactions with the highest number of co-citations first. To minimize falsely implied connections between genes and increase the reliability of the networks, we applied the ‘function word’ filter recommended by Jensen et al. [28] in which edges are only drawn between genes if the sentence linking them explicitly implies a functional interaction. For example, gene x ‘inhibits’ , ‘phosphorylates’ , ‘is the target of’ , gene y, etc. Finally, because gene-data loss occurs when converting ESTs to official bee genes, genes to fly homologs, and finally at the level of co-citation in the literature, we applied a series of Chi-square tests for independence to determine whether the gene composition of networks at each stage of analysis were an unbiased sample of the original gene expression dataset.

### Within network analysis

For each network we first used the Universal Protein Resource UniProt; [44] to assign each network gene a cellular function (e.g., kinase, cofactor, etc.). Specifically, we queried the UniProtKB database (gene name AND organism: “*Drosophila melanogaster* [7227]”) for all genes and distinguished these types of protein products visually using graphical symbols based on what is assigned under “General Annotation” of each gene name.

Second, we analyzed our networks above the level of the gene, via enrichment analysis as implemented in FuncAssociate software [45]. Here, we used a Fisher’s exact test (with a Monte Carlo False Discovery Rate simulation; adjusted family-wise error rate α = 0.05) to determine the most common functions and pathways of each network. To do this, we first calculated the number of genes expected to have particular Gene Ontology (GO) functions for a random network of the same size, assuming the random network samples genes relative to their true frequency in the (*Drosophila*) genome. We then compared this null expectation to the actual number of genes observed for the same functions in each of our networks. If our inferred networks are biologically functional, then we expect an over-representation of genes for that GO function.

For each network we also identified the highest connected (‘hub’) genes, and plotted the degree distribution (see Additional file 1: Figure S1), where the degree is the number of connections per gene [29]. For the single most connected gene in each network, we verified its implied interactions by querying genes against the *Drosophila* Interaction Database (DroID version 2013_02 database, http://www.droidb.org). This database is searchable for experimental evidence from protein-protein interaction studies, genetic interaction studies, transcription factor-gene interaction studies, and miRNA-gene interaction studies, if any.

### Between-network analysis

Because the input studies to our meta-analysis are variable with respect to populations of bees, experimental detail, and even array platform (Table 1), we expect our co-citation networks to vary. As a proxy for topographical convergence between inferred networks, either between studies for a given worker age or within studies for a different bee age, we determined the number of genes found to occur in more than one network. We then noted whether these recurring genes comprised the highest connected genes of any networks.

**Table 1:** Studies included in the meta-analysis

**Study**	**Experimental platform**	**Tissue type**	**Age of workers**^**2**^	**DEGs**	**DEGs after conversion**	**Number of potential networks**
Grozinger et al. [23]	Wild type bees in cages with QMP^1^	Brain	1	287	181	4
			2	1080	469	
			3	1242	540	
			4	391	334	
Thompson et al. [9]	Anarchist vs. wild type bees in colony with live queen	Brain	4	20	13	2
		Abdomen		20	12	
Grozinger et al. [10]	Wild type bees in cages with QMP	Brain	10	221	103	1
Thompson et al. [8]	Anarchist vs. wild type bees in colony with live queen	Brain	4	7	2	2
		Abdomen		5	2	
Cardoen et al. [11]	Wild type bees in colony without queen	Whole body	18	1292	1077	1
Backx et al. [46]	Wild type bees in cages with QMP	Brain	4	564	338	4
			6	782	527	
			8	623	428	
			10	534	387	

We included a total of n = 14 meta-datasets that we sourced from the independently published studies listed. For each meta-dataset we provide summary information on the experimental platform, the type of tissue and the age of workers. We also provide the number of differentially expressed genes (DEGs) that we converted to fly homologs prior to network construction.

^1^QMP, queen mandibular pheromone; ^2^Days post-eclosion.

## Results

We included six studies that represent 14 different microarray experiments in our meta-analysis (Table 1). These studies are comparable in that they screen for genes differentially expressed as a function of worker ovary activation in the presence or absence of queen pheromone, and therefore generate data that is suitable input for our proposed network analysis. From the set of input studies we can now potentially construct networks that describe the interactomes within brain, abdominal or whole body tissues, and do so across a range of worker ages from 1-day to 18-days post eclosion. On average, 52% (1884 of 3625) of ESTs identified from cDNA microarrays corresponded to Official Gene Set bee genes. Of all bee genes, including those from oligo arrays, roughly 78% (4409 of 5675) had unambiguous fruit fly homologs, and 19% (830 of 4409) of these genes had sufficient co-citation data to be incorporated into networks. Summary statistics for these genomic data are provided in Additional file 2: Table S1.

### Networks from brain tissue analysis

**Figure 1:**
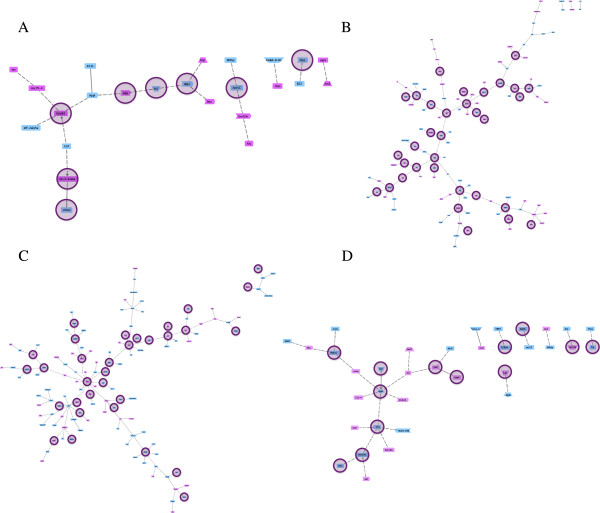
**Predicted co-citation networks from Grozinger et al.**[23]**gene lists for 1-, 2-, 3-, and 4-day-old workers (A-D, respectively).** Each node is a gene and each edge is a potential interaction between two genes. Pink genes are up-regulated in workers exposed to queen mandibular pheromone (as determined in the original study), and blue genes are correspondingly down-regulated. Genes highlighted with a circle are enriched for biological processes pertaining to ‘reproduction’ (GO:0000003).

**Figure 2:**
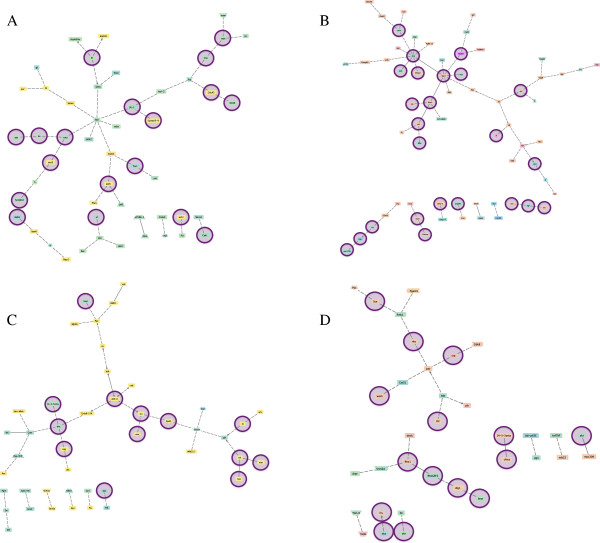
**Predicted co-citation networks from Backx et al.**[46]**gene list for 4-, 6-, 8-, and 10-day-old workers (A-D, respectively).** Description of symbols and topography is as for Figure 1, with the exception of a colour code change: genes ranging from yellow to pink are up-regulated in ovary in-active bees and genes ranging from yellow to green are down-regulated in ovary active bees.

From the 14 different data sets, we successfully generated 9 networks. The remaining five data sets from Thompson et al. [9], Thompson et al. [8], and Grozinger et al. [10] (Table 1) were not amenable to network analysis due to either the small number of DEGs identified (≤ 20 per experiment) and the even smaller subset that were suitable for downstream analysis via homology to the fly, or due to the small number of co-citations found in the literature. Eight out of nine networks were derived from worker brain tissue. Figure 1 shows the set of networks inferred from the DEG sets of Grozinger et al. [23], which correspond to workers of different ages. In each data set, we infer a single main network that incorporates a majority of genes, with only a minority of genes excluded from the main network to form minor connections among themselves, or to remain unconnected as singletons. Some networks reveal genes that are potentially of functional importance – for example, *dlg1* in Network 1C or *arm* in Network 1D are particularly well connected. From the Grozinger et al. [23] study, the networks we infer also vary in size, in this case ranging from n = 24-135 genes. The networks we infer from other brain tissue data sets showed comparable topologies. Figure 2 shows the networks derived from 4-, 6-, 8-, and 10-day old bees, as inferred from the DEG sets identified by Backx [46]. These networks vary in size from 34 to 63 genes and the highest connected genes include the immune-related transcription factor *Rel*[47] in Network 2A, the oogenesis related signaling protein *bsk*[48] in Network 2B, and *abd-A* in Network 2C, a transcription factor implicated in abdomen and gonad development [49].

### Network from whole body tissue analysis

**Figure 3:**
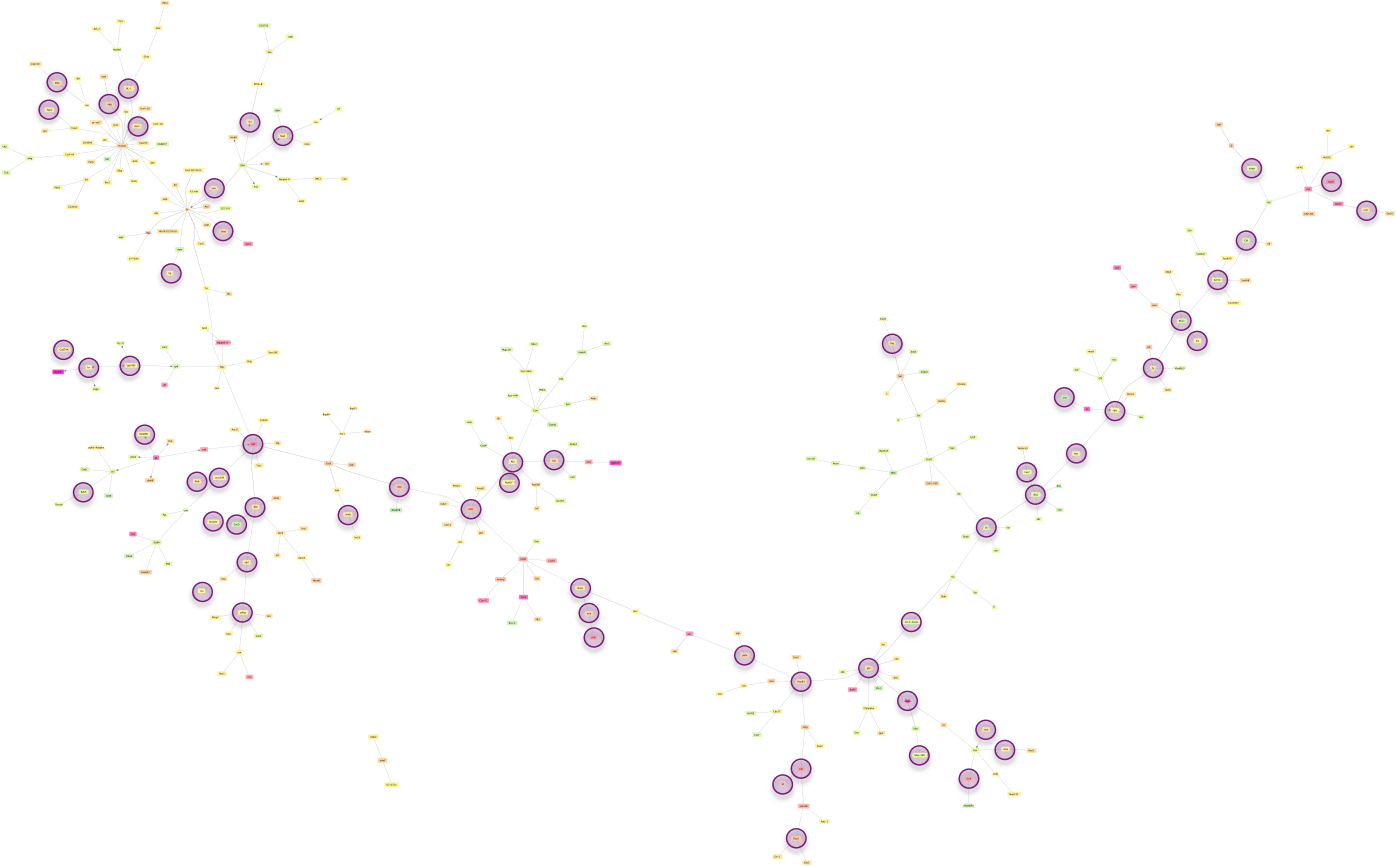
**Predicted co-citation network from Cardoen et al.**[11]**gene list for 18-day-old workers.** Description of symbols and topography is as for Figure 1, with the exception of a colour code change: genes ranging from yellow to green are up-regulated in ovary in-active bees and genes ranging from yellow to purple are down-regulated in ovary active bees.

The single experimental data set that was derived from whole body tissue (head + thorax + abdomen) yielded an expansive co-citation network, as expected given the tissue heterogeneity (Figure 3). This network corresponds to workers that are 18-days of age, and is inferred from the DEG set identified by Cardoen et al. [11]. This dataset corresponds to the oldest aged workers included in our meta-analysis, and the inferred main network consists of 323 genes with only three genes that remain disconnected. A *His2AV* gene (*His2Av*) is shown to have as many as 24 functional connections.

### Genes of functional importance

**Figure 4:**
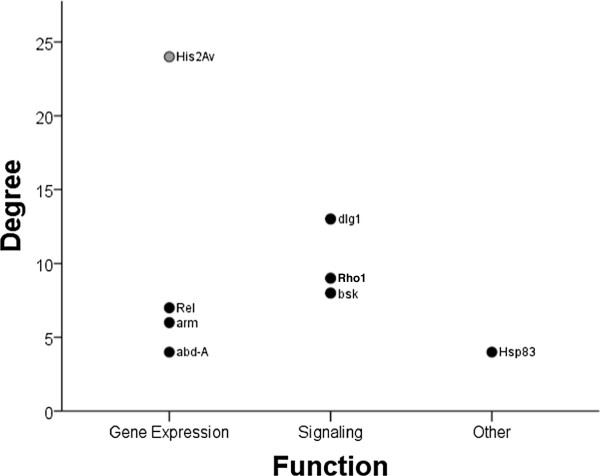
**Degree of the highest connected gene of each network.** Black hubs are from networks created with brain gene expression and grey hubs are from networks created with whole body gene expression.

Eight out of nine networks (all except Network 2D) contain highly connected genes that show between four (*Hsp83*; Network 1A) to twenty-four (*His2Av*; Network 3) interactions (Figure 4). GO analysis suggests that these so-called hub genes function to regulate gene expression (*Rel*, *abd-a*, *arm*, and *His2Av*), are involved in signaling (*dlg1*, *bsk*, *Rho1*), or are molecular chaperones (*Hsp83*). It is worth noting that six of these functionally important genes (*bsk*, *abd-A*, *Hsp83*, *Rho1*, *dlg1*, and *arm)* are implicated by GO analysis to function in reproduction (GO analysis is available as Additional file 3: Table S2). These highest connected genes are clearly relevant to our trait of interest, worker sterility.

In addition to co-citation support in the literature, we found experimental support for several of the hub gene interactions. DroID analysis confirmed that fully 32% of co-citation interactions (24 of 74) are associated with protein-protein interactions, transcription factor-gene interactions, genetic interactions, or combinations thereof, in flies and other model organisms (Summary of DroID analysis is available as Additional file 4: Table S3). All hub genes had at least one confirmed interaction, and one gene (*Hsp83*) had all of its four interactions experimentally confirmed. This level of cross-validation suggests that connections we have inferred from Pathway System analysis are biologically robust.

In general there was little overlap between the hub genes identified from our network meta-analysis and the most-differentially expressed genes prioritized from the individual microarray studies (see Additional file 5: Table S4). For example, the genes *Eip93F* and *Klp67A* (in Figure 3) and *Smr* (in Figure 2C) have large expression differences on the microarray, yet they tend to be found at the periphery of the networks with few connections instead of near the centre as highly connected hubs. Furthermore, highly connected hub genes such as *His2Av* (in Figure 3) and *dlg1* (in Figure 1C) were not exceptionally differentiated on the microarrays. Our network analysis has therefore identified a new list of candidate genes important to worker sterility that was previously overlooked by the array studies themselves.

### Network enrichment analysis

The networks inferred here show evidence for functional enrichment for genes related to multiple biological processes. Sixty terms were enriched in all networks, including oogenesis (GO:0048477), neuron differentiation (GO:0030182), and response to chemical stimulus (GO:0042221), among others (Table 2). These networks were also enriched for genes related to ‘reproduction’ (GO: 0000003), but even here the number and identity of these genes depended on the age of the bee and the type of tissue (Table 1). From this analysis, we reveal a total of 170 different genes involved in reproduction in our networks.

**Table 2:** Enrichment analysis of gene networks

**Category**	**GO term**	**GO number**	**NG**^**1**^	**Average O/E**
Ovary Activation	Reproduction	GO:0000003	250	3.06
	Female gamete generation	GO:0007292	171	4.40
	Oogenesis	GO:0048477	171	4.46
Brain and Behaviour	Neuron differentiation	GO:0030182	208	6.35
	Response to chemical stimulus	GO:0042221	194	4.55
Signaling	Signal transduction	GO:0007165	253	3.17
	Cell communication	GO:0007154	332	3.05
Foraging/Flight Related	Compound eye development	GO:0048749	109	4.83
	Locomotion	GO:0040011	141	5.02

For each network that we inferred (Networks 1-3) we found the GO terms that are most likely to represent the network’s biological functions. Sixty biological processes were significantly enriched among all nine networks (*P* < 0.01). Nine selected processes, the number of genes associated with these processes, as well as the observed/expected ratios averaged over all networks, are presented.

^1^The total number of genes found in all networks associated with the GO term.

Several pathways were also enriched across multiple networks. All but one network (Network 2C) were enriched for functional constituents of the ‘cell surface receptor signaling pathway’ (GO:0007166). Three (Network 2A, 2B, 3) were enriched for ‘insulin receptor signaling pathway’ (GO:0008286), one (Network 2C) for ‘dopamine receptor signaling pathway’ (GO:0007212), and one (Network 1B) for ‘steroid hormone-mediated signaling pathway’ (GO:0043401).

### Gene overlap among networks

There was visually little gene overlap among the networks, and no single gene was found in all nine networks. Yet, a significant proportion of genes (136 of 824, or 17%) were found in more than one network, with 10 of these genes found to span all four networks inferred from the Backx [46] study. Moreover, we found 34 genes to span all four networks inferred from the Grozinger et al. [23] study. In total, we found 96 genes to span at least two networks across all of the different studies. The most recurring genes, *Src42A* and *Hsp83*, were found in five of the nine networks. The hub genes of four networks were also re-occurring genes; *Hsp83* is found in five networks, *Rho1* and *dlg1* in four, and *Abl* in two.

### Co-citation network bias

The networks derived in this study vary in size. Network size is correlated with DEG list size (r = 0.827, *P* < 0.01). Other factors that influence network size include the proportion of DEGs with fly homologues, and the proportion of these with co-citation information currently available in PubMed. A majority of networks (6 of 10) showed no statistical bias with respect to proportion of genes up- or down-regulated, compared to the original data sets from which the networks were inferred (*χ*^2^ tests, *P* > 0.05 in each case). The remaining networks did however include a disproportionate number of genes up-regulated (*χ*^2^ = 13.58-24.4, d.f. = 3, *P* <0.001 in all cases), either upon conversion to fly homologues (Networks 1B) or upon retrieving co-citation links from PubMed (Networks 1D, 3 and 4). This biased sampling of some DEG sets simply reflects the information currently available within the PubMed database.

## Discussion

In this study we have generated the first gene networks that describe variation in ovary activation among honey bee workers. As such, these networks may be useful for understanding how worker reproductive altruism and sterility is regulated at the physiological and molecular level. The networks presented are derived from a functional analysis of gene sets previously identified from microarray studies (Table 1). One pattern to emerge from this meta-analysis is that each network is generally inclusive, with a majority of annotated genes forming linkages into single, main networks (Figures 1, 2, 3). This level of connectivity, together with the substantial size of some networks, suggests that the underlying DEG sets are biologically informative, and that the networks do reflect functionally interacting genes [50]. Moreover, each network, though highly variable in terms of gene membership, is enriched for biological processes related to reproduction, including oogenesis and other functional terms that are consistent with reproduction and reproductive regulation (Table 2). Our meta-analysis has therefore yielded a set of graphical hypotheses that potentially describe adaptively complex and biological functional networks that worker bees use to regulate personal reproduction within a social context.

### Co-citation networks

The eight networks derived from brain tissue varied substantially in gene membership, size and topology. The smallest network (n = 24 genes) describes how workers that are very young, only 1-day post eclosion, respond to queen pheromone at the molecular level (Figure 1A). This network is neither fully connected nor very large, but does show balanced expression between genes up- or down-regulated, and does identify a single most-connected gene that encodes a heat-shock protein (*Hsp83*). Bees this young may therefore show insufficient gene activity in response to pheromone to meaningfully assemble this activity into a functional network. Nonetheless, our identification of *Hsp83* is significant because this gene has previously been singled-out in relation to honey bee caste differentiation [51,52], oogenesis [53], and is a marker for changes in queen reproductive status [10,54]. All of these implied changes reflect a potential role in reproduction and reproductive divisions in labour.

Bees just one and two days older, however, show evidence of massively coordinated expression (Figures 1B, 1C). These co-citation networks are markedly more complex, and thus are more informative with respect to inferring function and identifying hub genes. The bias against up-regulated genes in this network suggests that the original gene set contained important elements to reproduction, but that have no GenBank homologue in the fly. The highest connected gene in Network 1B is *Rho1*, a protein that regulates signaling pathways in development [55]. Another highly connected gene in this network is *Abl*, which functions in neural development in honey bees and influences behavioural maturation [56]. Finally, caged 4-day old workers, at the age just prior to ovary activation [9], yield a relatively small co-citation network (Figure 1D). Here, the highest connected gene *arm* encodes a transcriptional activator in the wnt pathway to regulate the expression of many genes [57].

Figure 2 shows the four networks inferred through co-citation analysis of genes identified by Backx et al. [46]. The 4-day old bee co-citation network (Network 2A) is comparable in size to that inferred from Grozinger et al.’s [23] study (50 vs. 35 genes), but shared only one gene in common (*sima*). The highly connected genes *bsk* and *abd-A* have previously been implicated in reproduction in honey bees [48] and *Drosophila*[49] and their position as hubs suggests that they may be functioning similarly in our networks. The role of a central immune gene (*Relish*) in Network 2A is unclear, but other honey bee studies have also found immune genes to be differentially expressed between reproductives and non-reproductives [10,58], and networks modeling honey bee behaviour also contain transcription factors that code for immune genes as major hubs [59].

Figure 3 shows the single network inferred through co-citation analysis of genes identified by Cardoen et al. [11]. This network depicts a putative molecular mechanism through which the oldest workers included in our meta-analysis turn their ovaries *on* and *off* in response to pheromonal cues. This is the only co-citation network included in our meta-analysis that is derived from whole body tissue, as opposed to brain tissue (Table 1). This network is well connected, with only three genes separate from the main component, suggesting that the majority of these genes are indeed functioning together. This network showed some bias towards up-regulated genes in comparison to the initial DEG list, again during the detection of co-cited genes. The gene *His2Av* is not obviously related to reproduction but we have identified this gene silencer as a very well connected gene (24 interactions) and thus may be important to sterility. This gene is connected to those directly implicated in reproduction, including *eggless*. The gene *eggless* has a role in *Drosophila* oogenesis [60], so the identity of *His2Av* and its neighbours within our co-citation network for worker ovary activation and sterility warrants further attention.

### Highest connected genes

Candidate genes identified through microarray analysis highlight those that are highly differentially or chronically expressed, whereas those identified through network analysis instead feature genes that are highly connected. In our networks, the highly differentially expressed genes tend to have few connections and perhaps serve as the end result genes that directly affect worker phenotype. Highly connected hubs, on the other hand, tend to function early on in biological pathways as the initial genes that co-ordinate the expression of several other downstream target genes [61]. We see this feature in our networks; all but one hub gene (*Hsp83*) have functional roles involved in or upstream to gene expression (e.g., the transcription factors *Rel* and *abd-A* and the signaling proteins *Rho1* and *bsk*[59]).

### Testing candidate pathways for ovary activation

The networks generated in this study provide an opportunity to test previous ideas on the make-up of pathways for ovary activation in honey bees. One pathway that has been implicated in reproductive regulation is the insulin/insulin-like signaling (IIS) pathway. The IIS pathway acts upstream of ecdysteroid and juvenile hormone regulation to control solitary insect reproduction [62] and is required for insect vitellogenesis [63]. In social Hymenoptera, it appears to have a critical influence in the evolution of eusociality, as it has been specifically implicated in both reproductive division of labour between castes [32,38] and age-related division of labour within the worker caste [64]. Our enrichment analysis has identified significant elements of the IIS pathway in some of our networks. Network 2A (adjusted *P* < 6.00E-03), Network 2D (*P* < 1.00E-03), and Network 3 (*P* < 7.00E-03) were enriched for ‘insulin receptor signaling pathway’ (GO:0008286). Six key genes involved in this pathway and its regulation were present in the three networks, including the ligand *Ecdysone-inducible gene L2*, the receptor *chico*, the signaling molecules *Pten*, *dock*, and *Tsc1*, and the target transcription factor *foxo*[65]. This representation of genes involved throughout the entire pathway, as well as the enrichment across networks from different studies suggests the IIS pathway is strongly implicated in the control of worker sterility.

In addition we have identified elements of two other pathways, the dopamine receptor signaling pathway (GO:0007212, adjusted *P* <0.001) and the steroid hormone mediated signaling pathway (GO:0043401, adjusted *P* <0.01). The dopamine pathway has been implicated in caste differentiation [66] and ovary inactivation in the presence of QMP [67]. Honey bees have three dopamine receptors, *Amdop1*, *Amdop2*, and *Amdop3* expressed in their brains and ovaries. The genes *DopR* and *DopR2* were present in Network 2B and correspond to the *Amdop1* and *Amdop2* dopamine receptors. Workers of different ages and behavioural repertoires vary in their expression of all three dopamine receptors in response to queen pheromone [68]. The homovanillyl alcohol (HVA) component of QMP, one of the principle cues that induced worker sterility via ovary inactivation, binds to the *Amdop3* receptor [69], but QMP also modulates the expression of the other two receptors indirectly [67]. Moreover, HVA up-take is accelerated as workers transition between reproductive and non-reproductive states [70,71], suggesting that dopamine signaling is involved in reproductive response thresholds.

Finally, ecdysteroids involved in the steroid hormone mediated pathway have been implicated in bee brain function and oogenesis [13]. Network 1B contains an ecdysone receptor (*Ecr*) and the ecdysone-induced proteins *Eip78C* and *Hr46*[72]. *Ecdysone receptor* is needed for ovarian differentiation in *Drosophila*[73]. Ecdysteroids are therefore also potentially important to ovary signaling, consistent with Wang et al. [12] who demonstrated that *Ecr* is expressed in queen and worker ovaries, and *Hr46* affects female ovary size. Network 1B is derived from some of the youngest workers included in our analysis (2-days old). If steroid hormone pathways are important to ovary activation, then they would appear to act very early, prior to the visual development of ovaries.

Insulin, ecdysteroid and dopamine signaling pathways have been previously implicated in honey bee [32,65,74] or insect [75,76] reproduction, but our study is the first to statistically test their functional presence via gene representation within empirically derived networks. Our convergence onto these three pathways with known reproductive function suggest that *Apis* employs a mechanism similar to that used by unrelated non-social insects, up-holding a major prediction of the so-called reproductive ground plan hypothesis.

### A single conserved network for ovary activation?

At first glance, the networks derived here appear to be variable in gene composition, hub gene identity (Figure 4), and the overall interactions they describe (Figures 1, 2, 3). This apparent lack-of-convergence onto a single co-citation network, despite them being inferred from essentially similar datasets (Table 1) suggests that honey bee workers can use different networks to control personal reproduction, perhaps as a function of age, environmental circumstance, or both. Given that workers use different sensory modalities to interact with and respond to changes in their environment, including their social environment [58,77], it is conceivable that workers may use alternate or redundant pathways to control aspects of reproduction within colonies. Alternatively, a single pathway governing ovary activation in response to social cues seems more parsimonious [35,78]. If so, the multiple networks inferred here may represent segments of the larger, complete network that is still unknown. Different social and environmental signals may activate different suites of genes within this comprehensive network, explaining why the studies that vary in age, pheromone treatment, social structure, and environmental conditions have all captured different gene sets, with some degree of overlap.

## Conclusions

The networks identified here represent hypotheses for how workers regulate their ovaries to control reproduction. Within the context of their eusocial colonies, this reproductive machinery is of direct significance to sociogenomic theory that postulates the existence of ‘genes for altruism’ [79,80]. These genes have rarely been found [81] but the networks presented here, together with other efforts to describe how genes interact with each other and with their cellular, physical and social environment [12,26], provide a starting point from which we can begin to test ideas on the role of specific genes on reproductive phenotypes, including the prospect of finding genes for worker sterility. One approach might be to use functional genomic experiments that perturb hub genes or their neighbours, and then monitor worker phenotype. In particular it will be useful to measure how knock-outs affect worker phenotypes related to reproduction, including response to queen mandibular pheromone, ovary activation and egg laying, as well as other measures of social divisions in labour such as nurse-to-forager transitions or forager specializations. Finally, we suggest that future studies incorporate co-expression information and honey bee protein interactions, so that networks can then be made from *Apis* genes directly without the need for conversion to *Drosophila*, and would not rely on the somewhat haphazard availability of co-citation data in PubMed. Eventually it will then become possible to expand the networks beyond mRNA expression to incorporate regulatory and metabolome data and thereby provide an even more functional description of the mechanism that regulates ovarian physiology and reproductive altruism in honey bee workers.

### Availability of supporting data

The additional files supporting the results of this article are included within the article.

## Abbreviations

QMP: Queen Mandibular Pheromone; GO: Gene Ontology; DroID: *Drosophila* Interaction Database; IIS: insulin/insulin-like signaling; HVA: Homovanillyl alcohol.

## Competing interests

The authors declare that they have no competing interests.

## Authors’ contributions

GJT conceived of the study. GJT and EKM designed the study. EKM assembled and analyzed the meta data with input from MD. AGB provided data. All authors helped to draft the manuscript. All authors read and approved the final manuscript.

## Supplementary Material

Additional file 1: Figure S1 Degree distributions for each gene network and their relative R^2^ values. Click here for file

Additional file 2: Table S1 Gene loss occurring from converting original differentially expressed genes to those that appear connected in a co-citation network. Expressed sequence tags from microarrays are first converted to official bee genes. These genes are then converted to fruit fly homologs and entered into the co-citation analysis. Click here for file

Additional file 3: Table S2 Gene network enrichment analysis. Biological process GO term, the GO term ID, and the total possible genes associated with each GO term are presented. Where a network is enriched for a biological process, the corresponding *P* values, observed values, and expected values are shown. Click here for file

Additional file 4: Table S3 Edge confirmation by Drosophila Interaction Database. Confidence scores (where available) and evidence for Protein-Protein Interactions (PPI), Transcription Factor-Gene (PDI), and Genetic Interactions (GI) occurring between hub genes and their connected gene products in the various networks. Confidence scores range from 0-1. Interactions with higher values are more likely to be biologically relevant than interactions with lower values. Click here for file

Additional file 5: Table S4 A comparison of genes identified by network analysis and microarray analysis. Click here for file
