# Supplementary material for: Gene co-citation networks associated with worker sterility in honey bees
Source: BMC Syst Biol. 2014 Mar 26;8:38. doi: 10.1186/1752-0509-8-38 (PMC4030028; doi:10.1186/1752-0509-8-38)
Supplement: Additional file 1: Figure S1 — Degree distributions for each gene network and their relative R2 values. [file 1752-0509-8-38-S1.docx]

Network 3

Network 1D

Network 1A

Network 2B

Network 2A

**Additional file 1: Figure S1.** Degree distributions for each gene network and their relative R^2^ values.

Degree

Degree

Degree

Number of Genes

Number of Genes

Number of Genes

Network 2D

Network 2C

Network 1C

Network 1B
