# Supplementary material for: Gene co-citation networks associated with worker sterility in honey bees
Source: BMC Syst Biol. 2014 Mar 26;8:38. doi: 10.1186/1752-0509-8-38 (PMC4030028; doi:10.1186/1752-0509-8-38)
Supplement: Additional file 2: Table S1 — Gene loss occurring from converting original differentially expressed genes to those that appear connected in a co-citation network. Expressed sequence tags from microarrays are first converted to official bee genes. These genes are then converted to fruit fly homologs and entered into the co-citation analysis. [file 1752-0509-8-38-S2.docx]

**Additional file 2: Table S1**. Gene loss occurring from converting original differentially expressed genes to those that appear connected in a co-citation network. Expressed sequence tags from microarrays are first converted to official bee genes. These genes are then converted to fruit fly homologs, which are entered into the co-citation analysis.

| Study | Expressed Sequence Tags | % of ESTs that code for genes | Number of corresponding bee genes | % of bee genes with fruit fly homologs | Number of fruit fly homologs | % of homologs that make it into a network | Number of genes in network |
| --- | --- | --- | --- | --- | --- | --- | --- |
| Grozinger 2003 Day 1 | 268 | 79 | 213 | 85 | 181 | 13 | 24 |
| Grozinger 2003 Day 2 | 1125 | 50 | 560 | 84 | 469 | 23 | 110 |
| Grozinger 2003 Day 3 | 1224 | 48 | 590 | 92 | 540 | 25 | 135 |
| Grozinger 2003 Day 4 | 747 | 94 | 367 | 96 | 334 | 10 | 35 |
| Thompson 2006 - H | 20 | 65 | 13 | 100 | 13 | 0 | 0 |
| Thompson 2006 - A | 20 | 65 | 13 | 92 | 12 | 0 | 0 |
| Grozinger 2007 | 221 | 57 | 126 | 82 | 103 | 10 | 10 |
| Cardoen 2011 | N/A | N/A | 1293 | 83 | 1077 | 30 | 326 |
| Backx et al. 2012 Day 4 | N/A | N/A | 564 | 60 | 338 | 14 | 49 |
| Backx et al. 2012 Day 6 | N/A | N/A | 782 | 67 | 527 | 12 | 63 |
| Backx et al. 2012 Day 8 | N/A | N/A | 622 | 69 | 428 | 10 | 44 |
| Backx et al. 2012 Day 10 | N/A | N/A | 532 | 73 | 387 | 8 | 34 |
| **Average** | **N/A** | **52%** | **N/A** | **78%** | **N/A** | **19%** | **N/A** |
