# Supplementary material for: Gene co-citation networks associated with worker sterility in honey bees
Source: BMC Syst Biol. 2014 Mar 26;8:38. doi: 10.1186/1752-0509-8-38 (PMC4030028; doi:10.1186/1752-0509-8-38)
Supplement: Additional file 4: Table S3 — Edge confirmation by Drosophila Interaction Database. Confidence scores (where available) and evidence for Protein-Protein Interactions (PPI), Transcription Factor-Gene (PDI), and Genetic Interactions (GI) occurring between hub genes and their connected gene products in the various networks. Confidence scores range from 0-1. Interactions with higher values are more likely to be biologically relevant than interactions with lower values. [file 1752-0509-8-38-S4.docx]

**Additional File 4: Table S3.** Edge confirmation by Drosophila Interaction Database. Confidence scores (where available) and evidence for Protein-Protein Interactions (PPI), Transcription Factor-Gene (PDI), and Genetic Interactions (GI) occurring between hub genes and their connected gene products in the various networks. Confidence scores range from 0-1. Interactions with higher values are more likely to be biologically relevant than interactions with lower values.

| Network | Hub Gene | Degree in Networks | Number of Interactions in DroID | % of interactions confirmed by DroID | Associated Gene | Confidence Score | Evidence |
| --- | --- | --- | --- | --- | --- | --- | --- |
| Network 1A | Hsp83 | 4 | 4 | 100% | Appl | 0.315 | PPI |
|  |  |  |  |  | Hsf | - | PPI  PDI |
|  |  |  |  |  | eIF-2alpha | - | PPI |
|  |  |  |  |  | Hsc70-4 | 0.602 | PPI  PPI |
| Network 1B | Rho1 | 9 | 4 | 44% | tum | - | PPI |
|  |  |  |  |  | spir | - | PPI |
|  |  |  |  |  | Pkn | - | PPI  GI |
|  |  |  |  |  | Gdi | - | PPI |
| Network 1C | dlg1 | 13 | 4 | 31% | Pten | 0.34 | PPI |
|  |  |  |  |  | l(2)gl | - | PPI |
|  |  |  |  |  | Caki | 0.627 | PPI |
|  |  |  |  |  | Cam | 0.299 | PPI |
| Network 1D | arm | 6 | 1 | 17% | Src42A | - | GI |
| Network 3A | Rel | 7 | 1 | 14% | cact | 0.622 | PPI |
| Network 3B | Rac1 | 7 | 3 | 43% | Hsp83 | - | PPI |
|  |  |  |  |  | Rho1 | - | PPI  GI |
|  |  |  |  |  | bsk | 0.498 | PPI  GI |
| Network 3C | abd-A | 4 | 3 | 75% | cad | - | PDI |
|  |  |  |  |  | trx | - | PDI |
|  |  |  |  |  | hth | 0.412 | PPI  GI |
| Network 4 | His2AV | 24 | 4 | 17% | dom | 0.376 | PPI |
|  |  |  |  |  | Pc | - | GI |
|  |  |  |  |  | Parp |  | PPI |
|  |  |  |  |  | Top2 | - | PPI |
| **Average confirmed interactions** | **-** | **-** | **-** | **32%** | **-** | **-** | **-** |
