# Supplementary material for: Gene co-citation networks associated with worker sterility in honey bees
Source: BMC Syst Biol. 2014 Mar 26;8:38. doi: 10.1186/1752-0509-8-38 (PMC4030028; doi:10.1186/1752-0509-8-38)
Supplement: Additional file 5: Table S4 — A comparison of genes identified by network analysis and microarray analysis. [file 1752-0509-8-38-S5.docx]

**Additional File 5: Table S4.** A comparison of genes identified by network analysis and microarray analysis.

| Highest connected genes in network | Most differentially or chronically expressed on array |
| --- | --- |
| **Backx 2011** | |
| Rel  drk  bsk  Rac1  abd-A  Pax  Cam  pnr  dnc  Sin3A | Primo-2  CG4911  Apoltp  Piezo  Adc  Mct1  fz  Aats-his  kz  CG33170 |
| **Cardoen 2011** | |
| His2Av  Pc  Ubx  E2f  cdc2  CycB  phl  vas  Cam  Abl | wech  Eip93F  Fpps  yellow-g  hoe1  hkb  Klp67A  mael  dan  ac |
| **Grozinger 2003** | |
| Hsp83 | CG14168 |
| Rho1 | clt |
| EcR | Traf1 |
| Rpd3 | CG7474 |
| Lar | poe |
| dlg1 | frizzled2 |
| tkv | kr-h1 |
| WASp | Utx |
| arm | klumpfuss |
